# Telescoping into Adulthood: A Case Report of Intussusception in an Adult Patient

**DOI:** 10.21980/J8Q06C

**Published:** 2024-04-30

**Authors:** Neena Joy, Laura Kolster

**Affiliations:** * Morristown Medical Center, Department of Emergency Medicine, Morristown, NJ

## Abstract

Intussusception is a familiar diagnosis among the pediatric population; however, it is rarely considered among the adult population due to a myriad of life-threatening pathologies within the abdomen. We present an adult female who presented to the emergency department (ED) with abdominal pain and constipation. Laboratory testing and a computed tomography (CT) scan of the abdomen were ordered. Laboratory test results were notable for an elevated lymphocyte count as well as leukocyte esterase, white blood cells (WBC), and bacteria seen on urinalysis. The computed tomography scan detected a colo-colic intussusception secondary to a benign mass within the bowel lumen. The mass was surgically resected and the patient had an uneventful postoperative course. This unique case represents the occurrence of a pathology to which the adult population is not immune, and therefore should not be overlooked when evaluating a non-specific case of abdominal pain.

**Topics:**

Intussusception, colo-colic, obstruction, abdominal pain, constipation, female, mass, bowel, lymphocyte, ultrasound, computed tomography.

[Fig f1-jetem-9-2-v15] [Fig f2-jetem-9-2-v15]

**Figure f1-jetem-9-2-v15:**
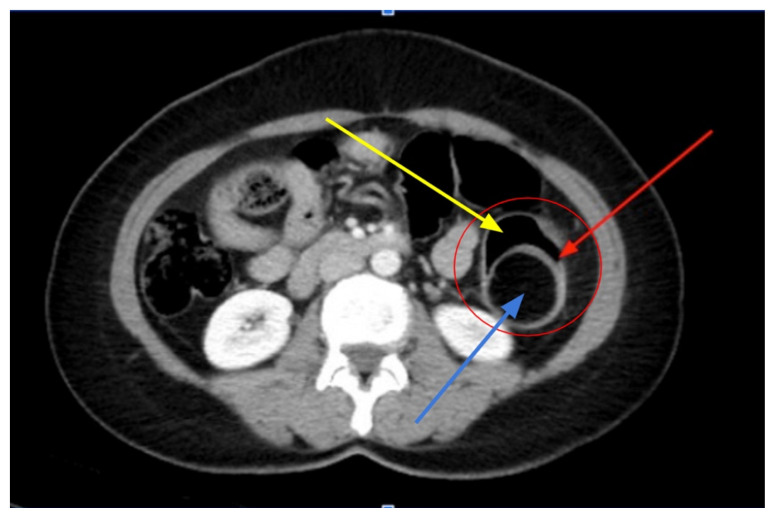


**Figure f2-jetem-9-2-v15:**
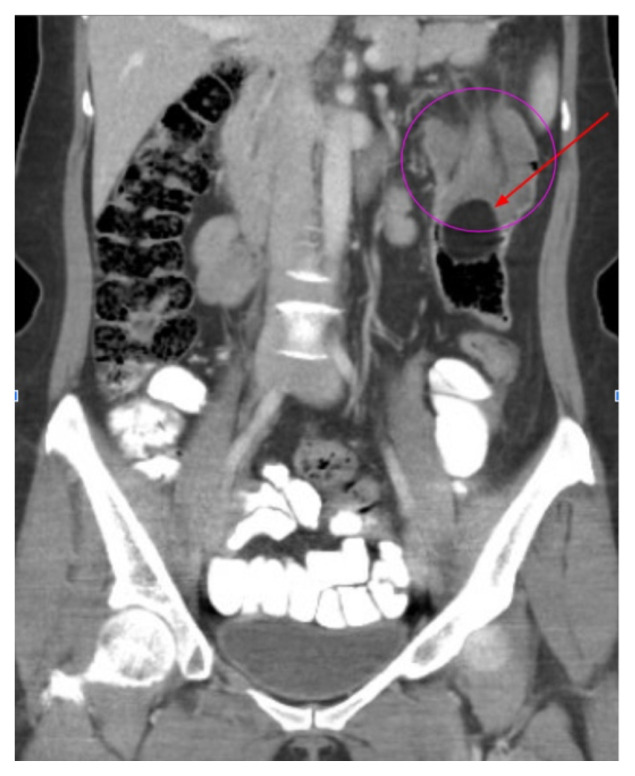


## Brief introduction

Intussusception is defined as the telescoping of one segment of bowel into an adjacent segment of bowel. This pathology presents with symptoms such as intermittent abdominal pain that fluctuates in severity, vomiting, bloating, and sometimes bloody bowel movements. It increases the risk of intestinal obstruction which can ultimately lead to ischemia, necrosis, perforation, and sepsis.^1^ Intussusception commonly occurs within the small bowel and targets the male pediatric population, typically within the age range of 6 to 18 months although cases can occur up to six years. Risk factors in children include viral and bacterial infections, older versions of the rotavirus vaccination, intestinal polyps, or cystic fibrosis due to altered intestinal motility.^1^ Despite this, a majority of cases of pediatric intussusception (PI) are of unknown etiology. On the other hand, intussusception is a rare occurrence in the adult population and reflects only 5% of all cases across all ages. In contrast to pediatric cases, adult cases present with pathologic lead points on imaging and as much as 90% of cases are secondary to neoplasms, which act as anatomical obstructions.^2^

## Presenting concerns and clinical findings

A 50-year-old female patient presented to the ED for evaluation of five days of abdominal pain. She initially attributed the pain to constipation; however, the pain progressed to the point where it was limiting her activities of daily living and ability to sleep. She reported having a bowel movement of normal color and consistency on the morning of her ED visit. Recently, she had an esophagogastroduodenoscopy (EGD) and colonoscopy performed and was diagnosed with Helicobacter Pylori gastritis, for which she was adequately treated.

She denied fevers, chills, sick contacts, nausea, vomiting, diarrhea, red or dark stools, chest pain, or shortness of breath. Her physical exam was notable for minimal epigastric tenderness upon deep palpation, but her abdomen was otherwise soft and non-distended with no guarding or rebound tenderness. Differential diagnoses included but were not limited to small bowel obstruction, acute coronary syndrome, pancreatitis, cholecystitis or biliary colic, or gastritis. The patient was treated with a bolus of intravenous fluids at one liter over 60 minutes; at that time her pain was not severe enough to require analgesics. She was told to restrict oral intake while anticipating the CT results. The patient’s vitals remained within normal limits throughout the course of her stay.

## Significant findings

Lab tests were notable for 40.3% lymphocytes seen on complete blood count, moderate leukocyte esterase with 5–10 WBCs/hpf, and few bacteria seen on urinalysis. Computed tomography imaging of the abdomen and pelvis with intravenous and oral contrasts was obtained. In the axial view, one will see a concentric ring formed by layers of bowel, mesenteric vessels, and fat (red arrow and circle); this is the equivalent of the ultrasonographic “target sign.” The inner ring (blue arrow) represents the lead point causing telescoping of the bowel. One can see that the proximal bowel is dilated (yellow arrow). In the coronal view, one can see an obstructive mass, also known as the lead point (red arrow), located in the lumen of the descending colon. Located proximal to the lead point are dilated loops of bowel with edematous changes and fat stranding (pink circle). The proximal portion of the bowel will take on a concentric appearance with the telescoping loop of bowel.

## Patient course

The general surgery attending was consulted and recommended a repeat CT scan in order to assess whether the oral contrast passed the site of intussusception. The repeat scan showed that the contrast indeed passed the site of intussusception, lowering concern for a large bowel obstruction. As the patient was hemodynamically stable and minimally symptomatic, the plan was made for discharge and to perform outpatient surgery within a few days.

Four days later, a general surgeon performed a laparoscopic left colectomy with laparoscopic splenic flexure mobilization and hand-sewn colo-colonic anastomosis. Per the operative note, the surgeon found a 3.8 cm submucosal lipoma with a focal ulcer and ischemic changes in the overlying mucosa. There was also mild serositis with serosal fibrosis and adhesions, but the pathology of the obtained tissue was negative for dysplasia or malignancy. The postoperative course was unremarkable, and the patient was subsequently discharged to home without complications.

## Discussion

Intussusception requires two key components: peristaltic contraction and a lead point.^3^ Etiologies of pediatric intussusception are often benign and most commonly idiopathic. In children, the classic symptom triad is intermittent abdominal pain, bloody or “currant jelly” diarrhea, and a palpable tender mass on physical exam.^3^,^4^ This triad is not seen in the majority of pediatric cases, and is most certainly not seen in adult cases. A majority of the cases occur between the ileum and the cecum and management involves barium or air enemas. Surgical resection is less common in the pediatric population.^3^

In contrast, adult intussusception (AI) only accounts for about 5% of all cases among all ages.^4^ Patients often present with nonspecific abdominal pain that fluctuates in severity, although occasionally the pain may be accompanied by nausea, vomiting, diarrhea, or blood in stool.^4^ Unfortunately, the adult chief complaint of abdominal pain is vague and is associated with a myriad of differential diagnoses that can make identifying intussusception rather difficult.

Diagnosis can be achieved with ultrasound, CT scan, or barium enema, although in the adult population, the first two options are the most popular and more sensitive tests. If a patient presents with a palpable abdominal mass, utilizing POCUS (point-of-care ultrasound) can have more than 90% sensitivity in diagnosing AI, although this may be affected by body habitus or the skill level of the ultrasonographer.^2^ Ordering a CT for diagnosis can have between 58–100% sensitivity and 57–71% specificity.^5^ On CT, you will expect to find all three layers of the bowel, possibly in addition to mesenteric vessels and fat, telescoping into an adjacent segment of bowel with surrounding edema.^6^ A lead point such as a malignant lesion, polyp, stricture, or diverticulum will likely be visible near the affected bowel. The segment may appear as a sausage-shaped mass when looking along its longitudinal axis; however, a cross-sectional view of the bowel segment may reveal a target-like or bull’s eye-lesion. This cross-sectional finding would be expected on an abdominal ultrasound exam as well.^3^

Between 75–90% of cases of AI have been diagnosed with an evident lead point, a majority of which are caused by malignant neoplasms.^7^ Based on a meta-analysis of 40 retrospective case series, colonic intussusception only comprised 19.9% of adult cases and among those cases, 46.5% had diagnostic testing that revealed a malignant tumor as the lead point.^7^ In contrast with the pediatric population, management of AI largely involves surgical exploration and resection, especially if malignancy is highly suspected as the etiology.^6^

In conclusion, our patient presented with a case of intussusception involving the descending colon and caused by an intraluminal benign mass. Adult intussuception, especially that of our patient, is a rare and difficult diagnosis that is often overlooked in the acute setting. However, it is important to identify and address this condition as soon as possible in order to prevent further complications.

## Supplementary Information








