# Supplementary figures and images for: Telescoping into Adulthood: A Case Report of Intussusception in an Adult Patient
Source: J Educ Teach Emerg Med. 2024 Apr 30;9(2):V15–7. doi: 10.21980/J8Q06C (PMC11068315; doi:10.21980/J8Q06C)

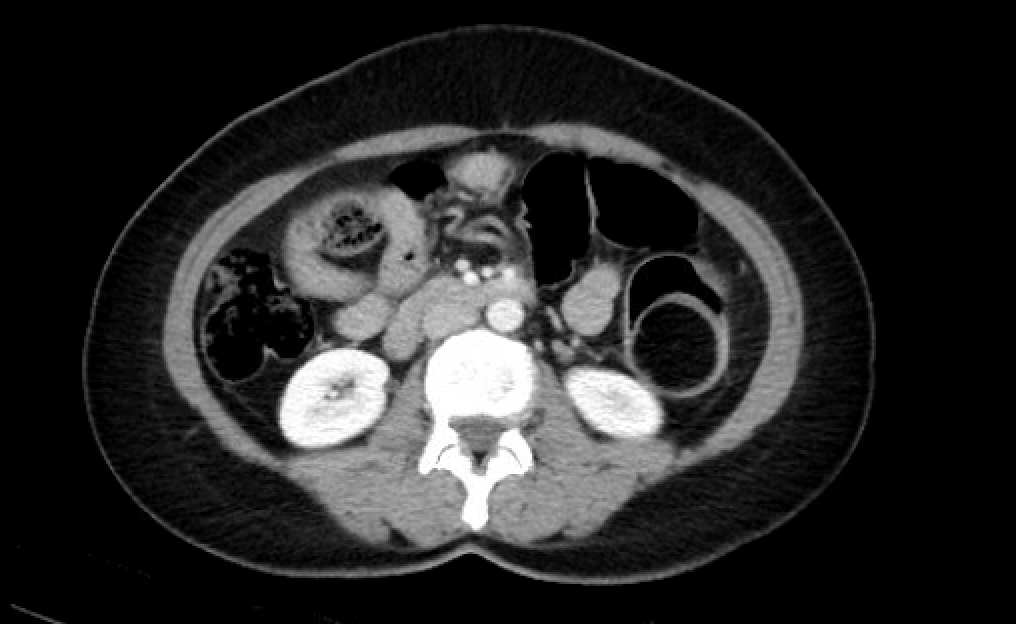

Supplement: Supplementary file 1 [file jetem-9-2-V15-supp1.jpeg]

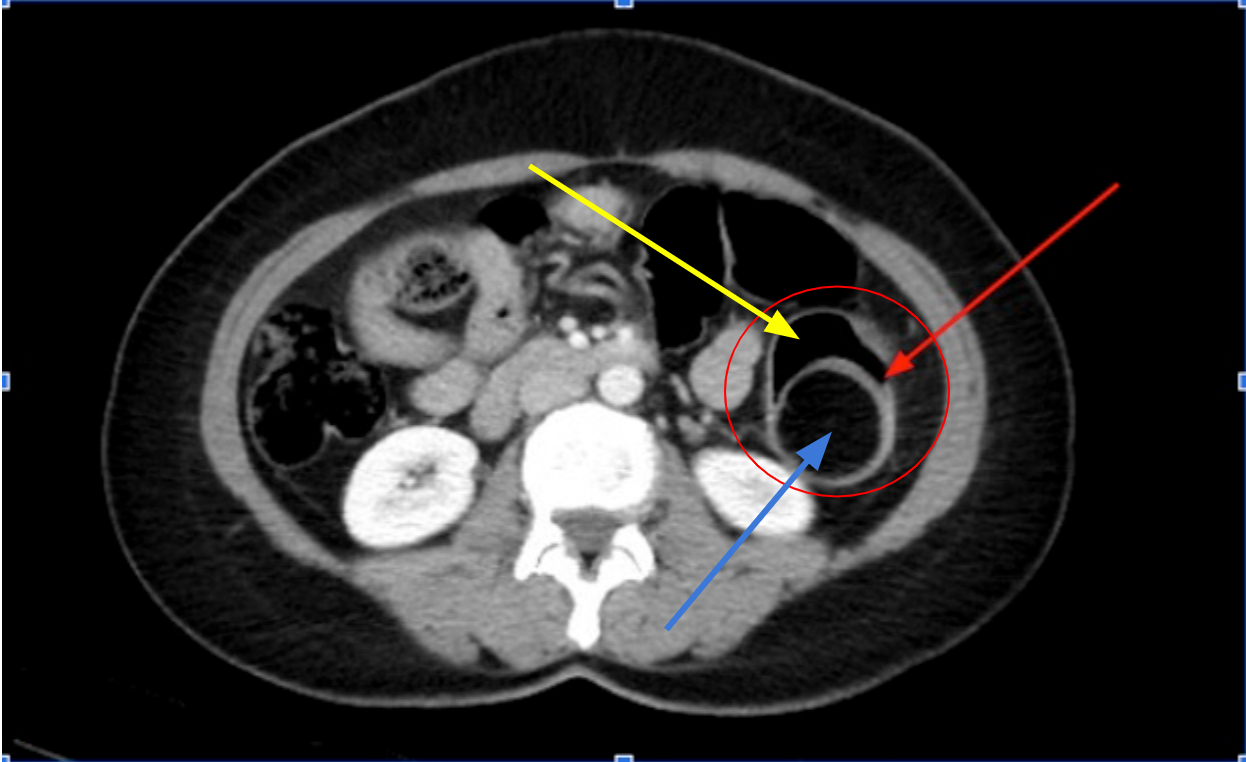

Supplement: Supplementary file 2 [file jetem-9-2-V15-supp2.jpeg]

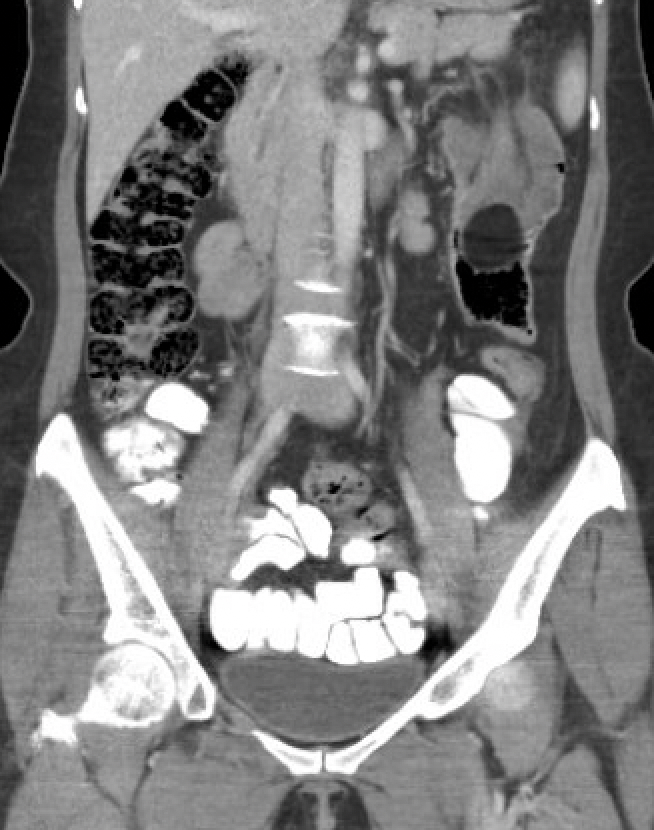

Supplement: Supplementary file 3 [file jetem-9-2-V15-supp3.jpeg]

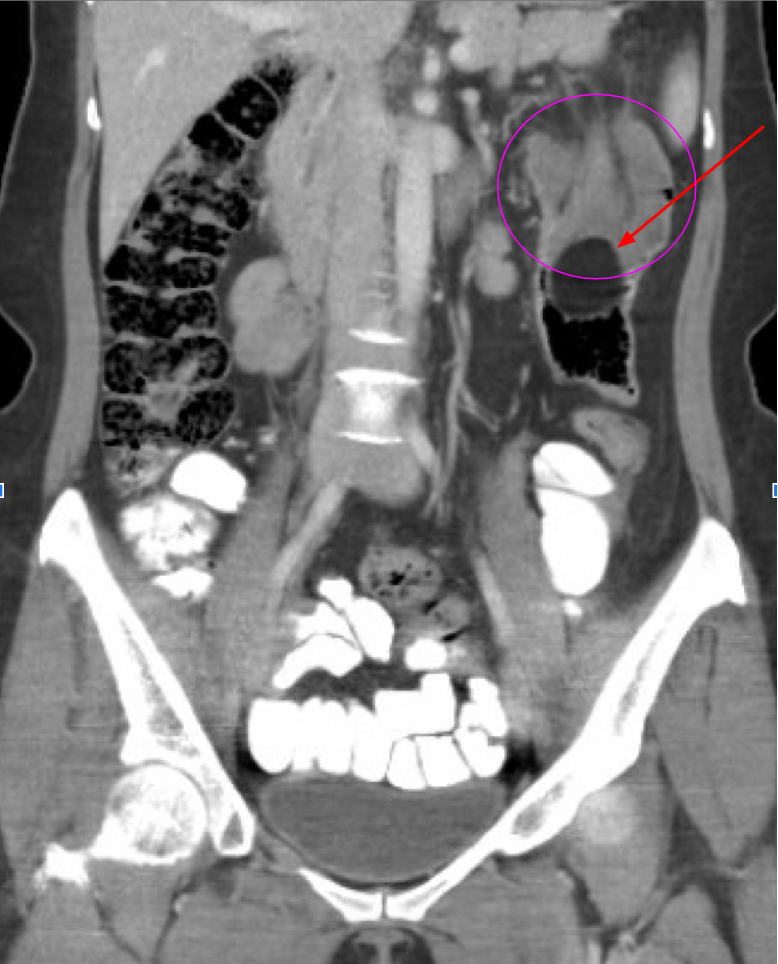

Supplement: Supplementary file 4 [file jetem-9-2-V15-supp4.jpeg]
